# Clinical features and efficacy of antiviral drug, Arbidol in 220 nonemergency COVID‐19 patients from East-West-Lake Shelter Hospital in Wuhan: a retrospective case series

**DOI:** 10.1186/s12985-020-01428-5

**Published:** 2020-10-23

**Authors:** Wei Gao, Si Chen, Kun Wang, Rongzhang Chen, Qian Guo, Jingjing Lu, Xiaodong Wu, Yanan He, Qiaoyun Yan, Shengyun Wang, Feilong Wang, Li Jin, Jing Hua, Qiang Li

**Affiliations:** 1 Department of Pulmonary and Critical Care Medicine, Shanghai East Hospital, Tongji University School of Medicine, Shanghai, 200123 China; 2 Department of Emergency and Critical Care Medicine, Changzheng Hospital, Second Military Medical University, Shanghai, 200082 China

**Keywords:** Coronavirus disease, COVID-19, Arbidol, Efficacy, SARS-CoV-2

## Abstract

**Objective:**

We aimed to describe the features of 220 nonemergency (mild or common type) COVID-19 patients from a shelter hospital, as well as evaluate the efficiency of antiviral drug, Arbidol in their disease progressions.

**Methods:**

Basic clinical characteristics were described and the efficacy of Arbidol was evaluated based on gender, age, maximum body temperature of the patients.

**Results:**

Basically, males had a higher risk of fever and more onset symptoms than females. Arbidol could accelerate fever recovery and viral clearance in respiratory specimens, particularly in males. Arbidol also contributed to shorter hospital stay without obvious adverse reactions.

**Conclusions:**

In the retrospective COVID-19 cohort, gender was one of the important factors affecting patient's conditions. Arbidol showed several beneficial effects in these patients, especially in males. This study brought more researches enlightenment in understanding the emerging infectious disease.

## Introduction

Since December 2019, an outbreak of unexplained epidemic pneumonia occurred in Wuhan, Hubei Province, China and has soon spread to the whole country. As of 6 May 2020, it spread to other 215 countries and a total of 3, 721, 393 globally laboratory-confirmed cases have been reported. On 11 February 2020, the novel epidemic disease was formally named as coronavirus disease 2019 (COVID‐19) and its causative virus as severe acute respiratory syndrome coronavirus 2 (SARS‐CoV‐2) [1]. In China, the number of COVID-19 infections has exceeded that of SARS population in 2002. In order to prevent the rapid spread of COVID-19, the Chinese government established 16 shelter hospitals for nonemergency patients (mild and common type). Within 35 days, Chinese doctors in these hospitals cured more than 10, 000 patients with a zero death. However, we haven't fully understood the clinical characteristics, disease evolution and therapeutic regime of COVID-19 patients in the special hospitals.

For the diagnosis of COVID-19, viral nucleic acid assay played a vital role by use of oropharyngeal swabs samples [2]. Comparatively, effective antiviral therapies seemed uncertain [2]. Arbidol, Oseltamivir, Ribavirin, Lopinavir/Ritonavir and Interferon (individual or combined treatment) were widely used in COVID-19 patients around China; however, none of them exhibited significant efficacy. We speculated that the results might partly attribute to the heterogeneity in study participants in the cohort under the pandemic. Among them, Arbidol was a broad-spectrum antiviral agent developed 30 years ago which could impair several steps within the life cycle of viruses, including attachment to cells and fusion with cellular membranes during virus entry. Herein, we described the features of 220 relatively mild COVID-19 patients from a shelter hospital, as well as evaluated the therapeutic efficiency of an antiviral drug, Arbidol.

## Methods

Our retrospective cohort from the East-West-Lake shelter hospital was composed of 220 laboratory-confirmed COVID-19 patients from 12 January 2020 to 2 March 2020. Approval for the retrospective analysis was obtained from the Ethics Commission of Shanghai East Hospital, China. The privacy rights of human subjects were protected all long.

The clinical features of different therapeutic groups were exhibited in Tables 1 and 2. A total of 130 patients received oral Arbidol at a dosage of 200 mg, 3 times a day for 4–8 days. Among them, 40 patients were given Arbidol and other antiviral drugs, including Oseltamivir (39 cases, 150 mg, 2 times a day for 4–8 days)/Ribavirin (1 case, 500 mg, 2 times a day for 4–8 days) while others were treated with Arbidol only. No antiviral drugs were given in 45 patients in control groups, and other antiviral drugs were given in 45 patients, including Oseltamivir (41 cases), Ribavirin (1 case), Ganciclovir (1 case, 500 mg, 2 times a day for 4–8 days), Prezcobix (1 case) or Oseltamivir + Ribavirin (1 case).

**Table 1:** Basic clinical and epidemic features based on Arbidol usage (two groups)

Feature	Without Arbidol (n = 90)	With Arbidol (n = 130)	*p* value
Age [years, M (IQR)]^a^	51 (44, 60)	48 (37, 56)	0.13
> 50 years (%)	52 (57.8)	59 (45.4)	0.07
Male (%)	47 (52.2)	72 (55.4)	0.64
Underlying disease (%)^b^	16 (17.8)	27 (20.8)	0.58
Onset symptoms			
Fever (%)	64 (71.1)	112 (86.2)	0.006
Less than 3 onset symptoms (%)^c^	52 (57.8)	82 (63.1)	0.43
Lab examination			
WBC [× 10^9^/L, M (IQR)]	5.2 (4.4, 6.1)	5.4 (4.3, 6.7)	0.17
Neutrophils % [M (IQR)]	66.5 (60.8, 70.2)	62.9 (55.3, 71.9)	0.22
Lymphocyte % [M (IQR)]	23.4 (19.1, 28.6)	23.4 (17.6, 32.1)	0.28
Platelet [× 10^9^/L, M (IQR)]	164.0 (132.5, 219.5)	166.5 (143.8, 211.3)	0.86
CT Findings			
Lung region distribution			
Unilateral (%)	2 (2.2)	1 (0.8)	0.36
Bilateral (%)	88 (97.8)	128 (98.4)	0.71
Absence of lesion (%)	0 (0.0)	1 (0.8)	0.4
Lesions feature			
GGOs^d^	37 (41.1)	65 (50)	0.19
Scattered patchy infiltration with interstitial alteration	39 (43.3)	32 (24.6)	0.004
Consolidation	2 (2.2)	7 (5.4)	0.24
More than one feature	12 (13.3)	25 (19.2)	0.25
Clinical types			
Mild type	0 (0.0)	1 (0.8)	0.4
Common type	90 (100.0)	129 (99.2)	0.4
Other treatments except for antiviral drugs			
Antibiotics	54 (60.0)	85 (65.4)	0.42
Chinese medicine (Lian Hua Qing Wen)	72 (80.0)	108 (83.1)	0.56
Methylprednisolone	1 (1.1)	1 (0.8)	1
Gamma immunoglobulin	1 (1.2)	2 (1.5)	1

^a^*M (IQR)* medium (inter quartile range)

^b^Including diabetes mellitus, high blood pressure, coronary heart disease, congestive heart failure, chronic kidney disease, chronic obstructive pulmonary disease, asthma, chronic liver disease, history of breast cancer, gout, rheumatoid arthritis and retrobulbar duodenal ulcer

^c^Including dry cough, expectoration, chest tightness and shortness of breath, fatigue, muscle soreness, stuffy running nose, headache, pharyngalgia, thoracalgia, dyspnea, phlegm blood, chills and poor appetite

^d^*GGOs* ground-glass opacity

**Table 2:** Basic clinical and epidemic features based on antiviral drugs usage (four groups)

Feature	Control (n = 45)	Other antiviral drugs (n = 45)	Arbidol only (n = 90)	Arbidol + other antiviral drugs (n = 40)
Age [years, M (IQR)]^a^	51 (40, 61)	52 (45, 59)	48 (36, 56)	51 (38, 58)
> 50 years (%)	25 (55.6)	27 (60.0)	39 (43.3)	20 (50.0)
Male (%)	22 (48.9)	25 (55.6)	52 (57.8)	20 (50.0)
Underlying disease (%)^b^	7 (15.6)	9 (20.0)	13 (14.4)	14 (35.0)
Onset symptoms				
Fever (%)	27 (60.0)	37 (82.2)	77 (85.6)	35 (87.5)
Less than 3 onset symptoms (%)^c^	29 (64.4)	23 (51.1)	57 (63.3)	25 (62.5)
Lab examination				
WBC (× 10^9^/ L, M (IQR))	5.3 (4.5, 6.2)	4.9 (4.3, 6.1)	5.4 (4.4, 6.5)	5.7 (4.1, 7.2)
Neutrophils % [M (IQR)]	65.5 (57.2, 70.3)	66.5 (61.5, 69.3)	62.8 (55.9, 71.1)	65.3 (54.4, 73.9)
Lymphocyte % [M (IQR)]	22.0 (16.9, 27.8)	24.6 (20.4, 29.1)	23.1 (17.6, 32.2)	24.2 (18.0, 32.7)
Platelet [× 10^9^/L, M (IQR)]	167.0 (143.0, 234.0)	160.0 (130.8, 201.8)	165.0 (142.5, 209.5)	179.0 (150.5, 232.5)
CT findings				
Lung region distribution				
Unilateral (%)	1 (2.2)	1 (2.2)	1 (1.1)	0 (0.0)
Bilateral (%)	44 (97.8)	44 (97.8)	89 (98.9)	39 (97.5)
Absence of lesion (%)	0 (0.0)	0 (0.0)	0 (0.0)	1 (2.5)
Lesions feature				
GGOs^d^	18 (40.0)	19 (42.2)	41 (45.5)	24 (60.0)
Scattered patchy infiltration with interstitial alteration	18 (40.0)	21 (46.7)	24 (26.7)	8 (20.0)
Consolidation	1 (2.2)	1 (2.2)	7 (7.8)	0 (0.0)
More than one feature	8 (17.8)	4 (8.9)	18 (20.0)	7 (17.5)
Other antiviral drugs				
Oseltamivir (%)	0 (0.0)	41 (91.2)	0 (0.0)	39 (97.5)
Ribavirin (%)	0 (0.0)	1 (2.2)	0 (0.0)	1 (2.5)
Ganciclovir (%)	0 (0.0)	1 (2.2)	0 (0.0)	0 (0.0)
Prezcobix (%)	0 (0.0)	1 (2.2)	0 (0.0)	0 (0.0)
Oseltamivir + Ribavirin (%)	0 (0.0)	1 (2.2)	0 (0.0)	0 (0.0)
Other treatments except for antiviral drugs				
Antibiotics	23 (51.1)	31 (68.9)	57 (63.3)	28 (70.0)
Chinese medicine (Lian Hua Qing Wen)	36 (80.0)	36 (80.0)	73 (81.1)	35 (87.5)
Methylprednisolone	0 (0.0)	1 (2.2)	1 (1.1)	0 (0.0)
Gamma immunoglobulin	1 (2.2)	0 (0.0)	2 (2.2)	0 (0.0)

^a^*M(IQR)* medium (inter quartile range)

^b^Including diabetes mellitus, high blood pressure, coronary heart disease, congestive heart failure, chronic kidney disease, chronic obstructive pulmonary disease, asthma, chronic liver disease, history of breast cancer, gout, rheumatoid arthritis and retrobulbar duodenal ulcer

^c^Including dry cough, expectoration, chest tightness and shortness of breath, fatigue, muscle soreness, stuffy running nose, headache, pharyngalgia, thoracalgia, dyspnea, phlegm blood, chills and poor appetite

^d^*GGOs* ground-glass opacity

For COVID-19 diagnosis, oropharyngeal swabs samples were collected for viral nucleic acid assay. The detection of SARS‐CoV‐2 nucleic acids in respiratory specimens was based on the final result and time. If the last two assays at an interval of at least 24-h were both negative, the viral nucleic acid negative conversion time was calculated using the first negative of the 2 consecutive negatives; otherwise, we considered it as not negative (including positive and suspected). Statistical analysis was performed using SPSS 17.0. Appropriate statistical methods were applied according to different data types.

## Results

Firstly, we described general characteristics of the retrospective cohort (Tables 1, 2). They were mild type (1 case) or common type (219 cases) COVID-19 patients according to the updated guidance [2]. Among them, male patients had a higher risk of fever than females (odds ratio (OR) = 2.47, 95% confidence interval (CI) 1.25–4.89, *p* = 0.01). They also tended to have more symptoms (≥ 3) than women (OR = 1.88, 95% CI 1.08–3.27, *p* = 0.03) (Additional file 1: Table S1). Based on the analysis, we concluded that gender might be a significant influence factor and should be taken into account when assessing the efficacy of Arbidol in the non-severe COVID-19 cohort.

**Fig. 1:**
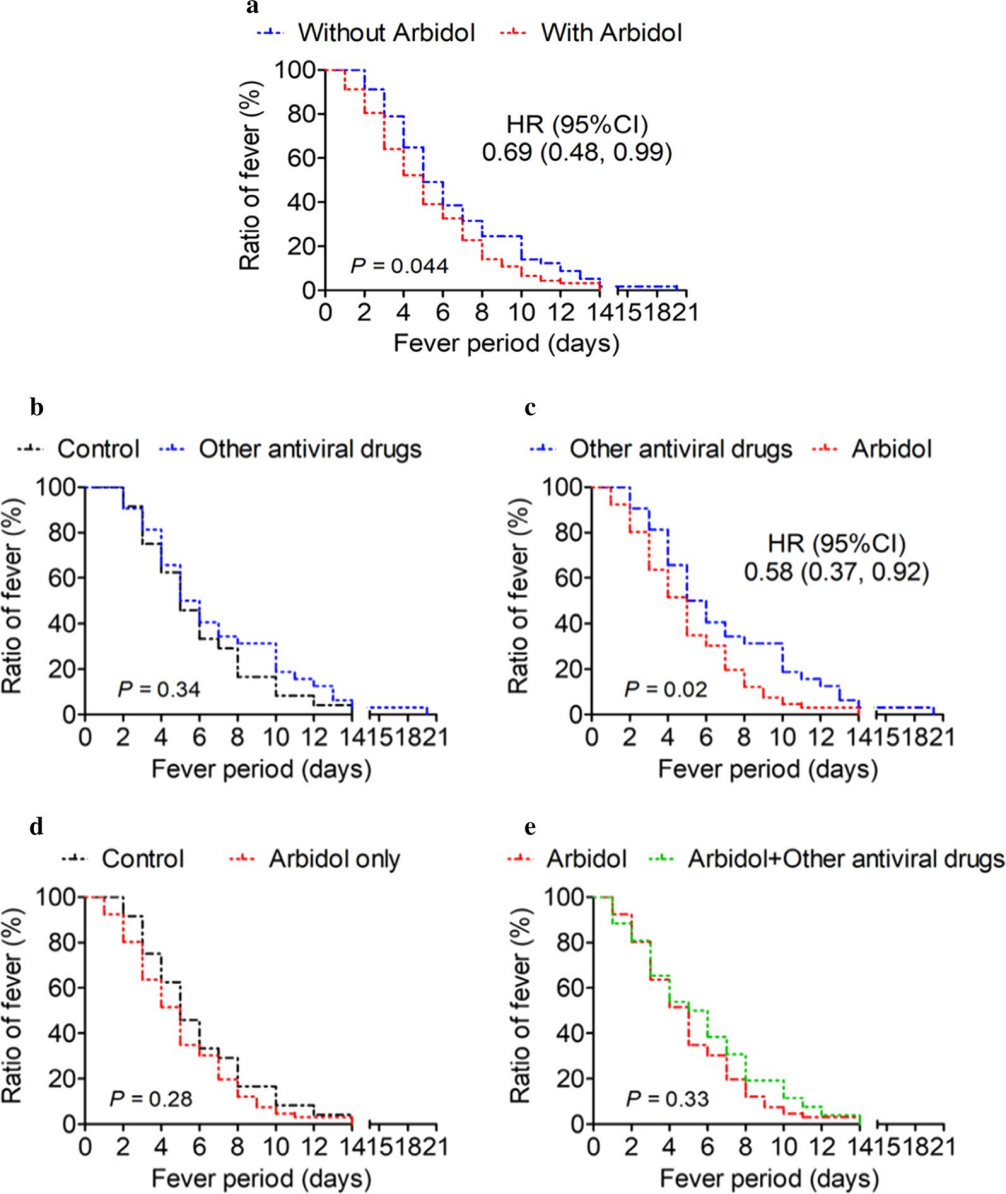
Time to fever resolution compared between distinct group based therapeutic strategies: **a** with and without Arbidol, **b** control and other antiviral drugs, **c** other antiviral drugs and Arbidol, **d** control and Arbidol only, **e** Arbidol and Arbidol combined with other antiviral drugs. *HR (95% CI)* hazard ratio (95% confidence interval)

**Fig. 2:**
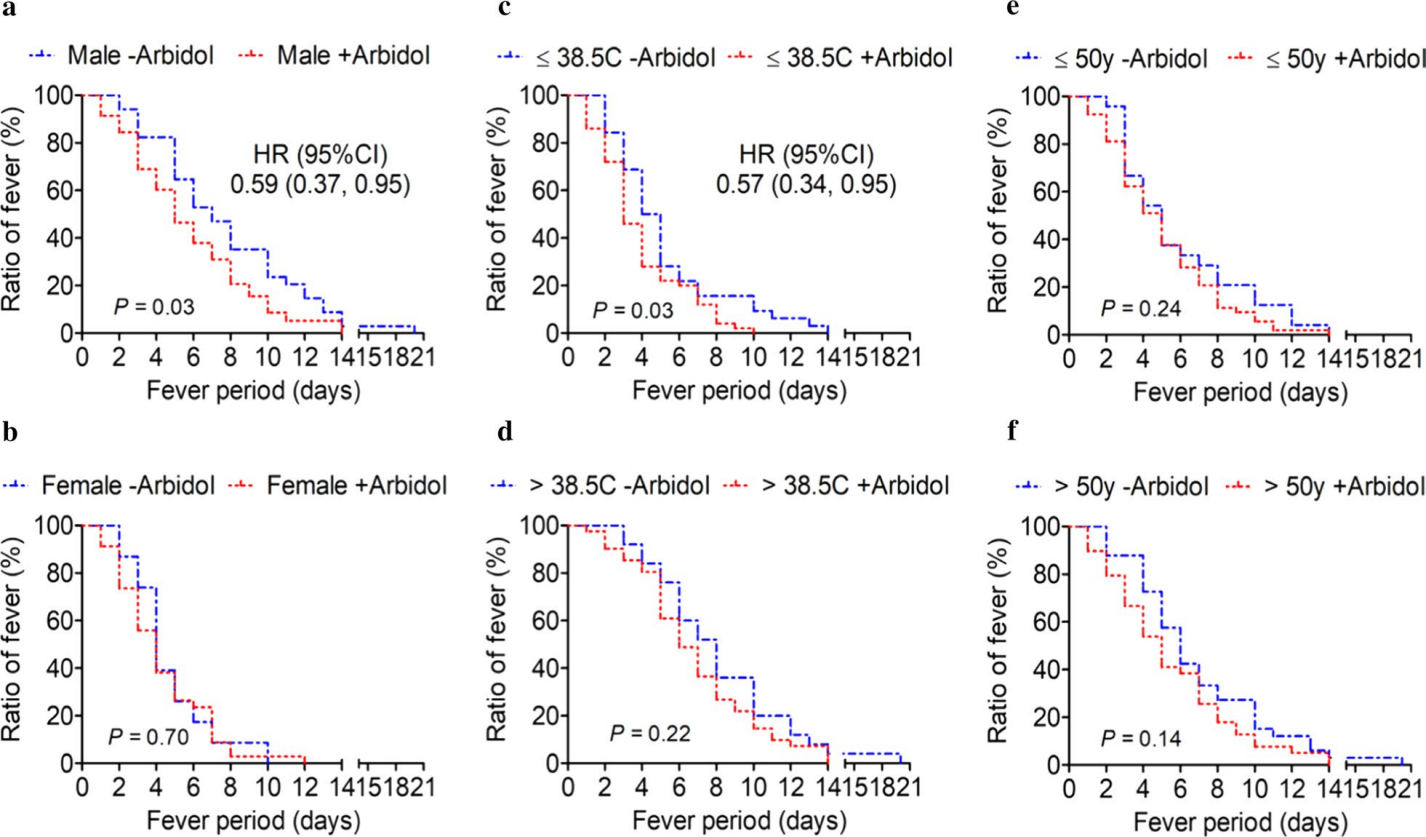
Time to fever recovery compared between distinct group based on gender (**a**, **b**), maximum body temperature (**c**, **d**) and age (**e**, **f**). *HR (95% CI)* hazard ratio (95% confidence interval)

Afterwards, we found that fever resolved more slowly in patients without Arbidol administration (hazard ratio (HR) = 0.69, 95% CI 0.48, 0.99, *p* = 0.02) (Fig. 1a). When subgrouping by therapeutic strategies, we only discovered the significant improvement of recovery time between other antiviral drugs (91.2% Oseltamivir) group and Arbidol group (HR = 0.58, 95% CI 0.37–0.92, *p* = 0.02) (Fig. 1c). Combination of Arbidol and other antiviral drugs (97.5% Oseltamivir) did not show better efficacy (Fig. 1e). Besides, in males and patients with lower-grade fever (≤ 38.5℃), Arbidol showed superior efficacy in fever recovery (HR = 0.59, 95% CI 0.37–0.95, *p* = 0.03; HR = 0.57, 95% CI 0.34–0.95, *p* = 0.03), which were not obvious in women and higher-grade fever subjects (Fig. 2a–d). Age was not the key point that affected Arbidol efficacy in this respect (Fig. 2e, f).

Subsequently, we observed that negative-converting rate of nucleic acid within 14 days in non-Arbidol group was lower than that of Arbidol group (OR = 0.47, 95% CI 0.24–0.91, *p* = 0.028). The effect of Arbidol was more remarkable when compared to patients without any antiviral drugs (OR = 0.23, 95% CI 0.10–0.57, *p* = 0.002). At the last assay, a total number of 14 patients still got non-negative results. In patients without Arbidol application, we saw higher non-negative rate compared with others (OR = 3.13, 95% CI 1.00–9.83, *p* = 0.049) (Table 3). Consistent with the above data, Arbidol showed obvious efficacy on viral clearance in males (OR = 0.27, 95% CI 0.11–0.66, *p* = 0.005 for negative-conversion within 2 weeks; OR = 8.40, 95% CI 1.70–41.42, *p* = 0.006 for not negative rate at last assay). In particular, we found that patients with not negative results in non-Arbidol group were all males, which was improved noticeably in Arbidol group (Table 4).

**Table 3:** Viral nucleic acid negative-conversion within 14 days and not negative rate at last assay in different treatment groups

Feature	Without Arbidol (n = 63)^a^	With Arbidol (n = 99)	*p* value	OR (95% CI)
Time to PCR negative in respiratory specimens				
≤ 14 days (%)	35 (55.6)	72 (72.7)	0.028*	0.47 (0.24, 0.91)
> 14 days (%)	28 (44.4)	27 (27.3)	0.028*	
Not negative (%)	9 (14.3)	5 (5.1)	0.049*	3.13 (1.00, 9.83)

	Control (n = 32)	Other antiviral drugs (n = 31)	Arbidol only (n = 69)	Arbidol + other antiviral drugs (n = 30)
≤ 14 days (%)	14 (43.8)	21 (67.7)	53 (76.8)*^b^	19 (63.3)
> 14 days (%)	18 (56.2)	10 (32.3)	16 (23.2)	11 (36.7)
Not negative (%)	6 (18.8)	3 (9.7)	5 (7.2)	0 (0.00)

*Statistically significant *p* vaues

^a^A minority of patients didn't accept nucleic acid detection after hospitalization

^b^Control group versus Arbidol group: OR = 0.23, 95% CI 0.10–0.57, *p* = 0.002

**Table 4:** Viral nucleic acid negative-converting rate within 14 days and not negative rate at last assay in patients with or without Arbidol based on gender, age and maximum body temperature

Male	Without Arbidol (n = 39)	With Arbidol (n = 58)	*p* value	OR (95% CI)
Time to PCR negative in respiratory specimens				
≤ 14 days (%)	19 (48.7)	45 (77.6)	0.005*	0.27 (0.11, 0.66)
> 14 days (%)	20 (51.3)	13 (22.4)	0.005*	
Not negative (%)	*9 (23.1)*	2 (3.4)	0.006*	8.40 (1.70, 41.42)

Female	Without Arbidol (n = 24)	With Arbidol (n = 41)	*p* value	OR (95% CI)
≤ 14 days (%)	16 (66.7)	27 (65.9)	1	
> 14 days (%)	8 (33.3)	14 (34.1)	1	
Not negative (%)	0 (0.00)	3 (7.3)	0.55	

≤ 50 years	Without Arbidol (n = 30)	With Arbidol (n = 59)	*p* value	OR (95% CI)
≤ 14 days (%)	16 (53.3)	43 (72.9)	0.096	
> 14 days (%)	14 (46.7)	16 (27.1)	0.096	
Not negative (%)	4 (13.3)	3 (5.1)	0.22	

> 50 years	Without Arbidol (n = 33)	With Arbidol (n = 40)	*p* value	OR (95% CI)
≤ 14 days (%)	19 (57.6)	28 (70.0)	0.33	
> 14 days (%)	14 (42.4)	12 (30.3)	0.33	
Not negative (%)	5 (15.2)	2 (5.0)	0.23	

≤ 38.5 C	Without Arbidol (n = 42)	With Arbidol (n = 67)	*p* value	OR (95% CI)
≤ 14 days (%)	24 (57.1)	47 (70.1)	0.22	
> 14 days (%)	18 (42.9)	20 (29.9)	0.22	
Not negative (%)	6 (14.3)	4 (6.0)	0.18	

> 38.5C	Without Arbidol (n = 21)	With Arbidol (n = 32)	*p* value	OR (95% CI)
≤ 14 days (%)	11 (52.4)	25 (78.1)	0.07	
> 14 days (%)	10 (47.6)	7 (21.9)	0.07	
Not negative (%)	3 (14.3)	1 (3.1)	0.29	

*Statistically significant *p* vaues

The medium hospital day in patients without antiviral drugs and treated with Arbidol was 19 and 15.5, respectively (*p* = 0.02). Considering the influence factors, we further demonstrated that Arbidol might contribute to the reduced hospitalization times in younger patients (≤ 50 year, *p* = 0.04) (Table 5). During our observation period, no obvious adverse reaction was noted in Arbidol treated patients. One case from Arbidol group presented with allergic skin rash due to Moxifloxacin and the medication had to be dis-continued.

**Table 5:** Hospitalization days in different treatment groups or in patients with or without Arbidol based on gender, age and maximum body temperature

Feature	Without Arbidol (n = 49)	With Arbidol (n = 72)	*p* value	OR (95% CI)
Hospital day [M (IQR)]	18 (16, 20)	17 (14, 21)	0.25	

	Control (n = 21)	Other antiviral drugs (n = 28)	Arbidol only (n = 48)	Arbidol + other antiviral drugs (n = 24)
Hospital day [M (IQR)]	19 (17, 21)	18 (14, 20)	15.5 (12, 20) *^a^	19.5 (17, 21)

Male	Without Arbidol (n = 24)	With Arbidol (n = 40)	*p* value
Hospital day [M (IQR)]	19 (14.5, 20)	16 (14, 20)	0.25

Female	Without Arbidol (n = 25)	With Arbidol (n = 32)	*p* value
Hospital day [M (IQR)]	18 (16.5, 20)	18 (13.3, 21)	0.75

≤ 50 years	Without Arbidol (n = 23)	With Arbidol (n = 49)	*p* value
Hospital day [M (IQR)]	18 (17, 20)	17 (13.5, 20.5)	0.04*

> 50 years	Without Arbidol (n = 26)	With Arbidol (n = 23)	*p* value
Hospital day [M (IQR)]	18 (14, 20)	18 (14, 21)	0.94

≤ 38.5C	Without Arbidol (n = 33)	With Arbidol (n = 51)	*p* value
Hospital day [M (IQR)]	19 (16.5, 20)	18 (14, 21)	0.23

> 38.5C	Without Arbidol (n = 16)	With Arbidol (n = 21)	*p* value
Hospital day [M (IQR)]	17.5 (14, 19)	17 (12.5, 20)	0.73

*Statistically significant *p* vaues

^a^Control group versus Arbidol group (*p* = 0.02)

## Discussion

Arbidol has been reported to have inhibitory effects on a diverse array of viruses such as influenza, Zika virus, respiratory syncytial virus, adenovirus, Coxsackie B5, parainfluenza, Ebola and hepatitis B and C viruses [3–7]. Mechanismly, it inhibited the fusion of influenza virus with endosomal membrane through binding to a hydrophobic cavity in the hemagglutinin on virus surface and stabilizing the pre-fusion conformation of hemagglutinin [5]. Owing to the broad-spectrum efficacy, Arbidol has been licensed for prophylaxis and treatment of acute respiratory infections, including influenza in China and Russia [5]. As for COVID-19, Chen et al*.* [8] found no difference between Lopinavir/Ritonavir and Arbidol in relieving symptoms or accelerating virus clearance. However, the subsequent multicenter, prospective research carried out by Wei et al*.* [9] demonstrated that the triple combination antiviral therapy of Arbidol, Lopinavir/Litonavir and recombinant interferon α-2b showed shorter viral shedding time and hospitalization time compared with the dual combination antiviral therapy without Arbidol. They also found that 10–30 μmol/L Arbidol effectively inhibited the coronavirus 60-fold compared with the untreated control group, as well as significantly alleviated the injury of SARS-CoV-2 to cells by chemosensitivity testing in vitro (Data were not published). Herein, we discovered the efficacy of Arbidol on viral shedding, thus accelerating disease relief in the nonemergency COVID-19 patients. We noticed that males displayed higher fever and more COVID-19 symptoms, which might due to the up-regulated SARS-Cov-2 receptor, angiotensin‐converting enzyme 2 (ACE2) by smoking and testosterone level, as well as excessive immune-inflammatory response [10, 11]. Furthermore, males exhibited better drug response, suggesting certain microenvironment (such as pH, ion, hormone and cytokines) might strengthen the efficacy of Arbidol. More studies in vivo and in vitro could be performed to identify the exact mechanisms.

Several results deserved explanations. Arbidol shortened fever duration compared with the patients without Arbidol and with other antiviral drugs respectively (Figs. 1, 2). The effect seemed more prominent when given early in the disease and in male patients. As shown in Fig. 1e, combination of Arbidol and other antiviral drugs did not show better efficacy compared to Arbidol only. We speculated the reduction in the first 5 days of fever period was mainly due to Arbidol and application of several antiviral drugs simultaneously might have aggravated adverse reaction or induced multiple adverse reactions. Additionally, all patients in Arbidol + other antiviral drugs group achieved negative nucleic acid in their respiratory specimens, but not in Arbidol only group (Table 3), though the difference was insignificant. We suggested that the number of subjects was not enough, therefore leading to certain inconsistency in the result.

Despite efforts to exclude bias by critical analysis, there were still several limitations in our research. First of all, this was a retrospective study and has not undergone rigorous clinical trial design. Therefore, it could not provide direct evidence for the effectiveness of Arbidol among COVID-19 patients. However, these results provided implications for further experimental or clinical researches on Arbidol usage and even guided the development of novel therapeutics against SARS-CoV-2. Secondly, during the early onset (from January to February, 2020) of the disease in this cohort, the treatment therapies might not accord with the latest guideline. These relatively mild patients were mainly administrated with Arbidol and/or Oseltamivir as antiviral treatments because of the rescuing urgency. In that case, we excluded the bias of antibiotic and traditional Chinses medicine which were widely used in our cohort (Tables 1, 2) and found the significant results of Arbidol. It should be noted that the efficacy might attribute to the combined effects, such as Arbidol combined with Chinses medicine. This deserved further studies in a larger cohort. Thirdly, the patients were hospitalized in shelter hospital (originated from gymnasiums, convention center and so on), where laboratory tests and chest CT could not be carried out promptly as a consequence of equipment and faculty deficiency. Therefore, our efficacy evaluation system was not perfect. Viral nuclei acid detection could not be performed everyday nor within 7 days since the first diagnosis; thus, we selected negative-conversion within 14 days as the indication of better drug response. This study aimed to bring more researches enlightenment in understanding the emerging infectious disease. Despite this, we also suggested the double-blinded randomized clinical trials on Arbidol application in COVID-19 patients, especially in mild and common type.

## Conclusion

In the retrospective COVID-19 cohort of 220 nonemergency patients form one shelter hospital, we analyzed and concluded that male patients had a higher risk of fever and more onset symptoms than females. Besides, Arbidol showed beneficial effects on fever recovery, viral clearance and shorter hospital stay in these patients, especially in males. Double-blinded randomized clinical trials to determine the most effective treatments for COVID-19 are still needed. Finally, we hope that human beings can soon overcome difficulties together in the “war” against COVID-19.

## Supplementary information

**Additional file 1: Table S1**. Basic clinical and epidemic features based on gender. a Including dry cough, expectoration, chest tightness and shortness of breath, fatigue, muscle soreness, stuffy running nose, headache, pharyngalgia, thoracalgia, dyspnea, phlegm blood, chills and poor appetite.

## Data Availability

The datasets used and/or analyzed during the current study are available from the corresponding author on reasonable request.

## Abbreviations

COVID‐19: Coronavirus disease 2019
SARS‐CoV‐2: Severe acute respiratory syndrome coronavirus 2
OR: Odds ratio
CI: 95% Confidence interval
HR: Hazard ratio
ACE2: Angiotensin‐converting enzyme 2
